# A Cross-Sectional Analysis of Pain, Neck Disability, Functional Performance, and Quality of Life in Patients with Cervical Spondylosis

**DOI:** 10.3390/jcm15010094

**Published:** 2025-12-23

**Authors:** Arbnore Ibrahimaj Gashi, Arjeta Azemi, Tine Kovačič

**Affiliations:** 1 Physiotherapy Department, Faculty of Medicine, University of Prishtina, 10000 Prishtina, Kosovo; arbnore.gashi@uni-pr.edu; 2 Physical Therapy and Rehabilitation Clinic “Physio-ANA”, 70000 Ferizaj, Kosovo; xhakliarjeta@gmail.com; 3 Physiotherapy Department, Faculty of Medicine, University Alma Mater Europaea—ECM, 2000 Maribor, Slovenia

**Keywords:** cervical spondylosis, neck disability index, patient-specific functional scale, McGill Quality of Life, range of motion, cross-sectional study, musculoskeletal rehabilitation, numeric pain rating scale

## Abstract

**Background/Objectives:** Cervical spondylosis is a cause of recurrent neck pain, disability, and poor quality of life (QoL). This study aimed to examine the relationships among pain intensity, neck disability, functional performance, body mass index, and quality of life in individuals with cervical spondylosis. **Methods:** The study was conducted as a cross-sectional study on 111 participants (33 males and 78 females). Data were collected using different assessment tools such as Neck Disability Index (NDI), Numeric Rating Scale (NPRS) for pain, Patient-Specific Functional Scale (PSFS), and McGill Quality of Life Questionnaire (Part A). Independent *t*-tests and ANOVA assessed group differences, whereas correlations and multiple linear regression examined the association of QoL and function. Data from 107 out of 111 participants were further analysed due to missing data. **Results:** No significant gender- or activity-based differences were observed for pain, disability, function, or QoL (*p* > 0.05). However, negative correlations were found to be significant between NDI and both PSFS (r = −0.41, *p* < 0.001) and QoL (r = −0.52, *p* < 0.001). Regression analysis identified NDI, pain intensity, BMI, and PSFS as significant independent correlates of QoL (Adj. *R*^2^ = 0.426, *p* < 0.001), although BMI alone was associated with functional ability (Adj. *R*^2^ = 0.141, *p* = 0.008). Higher neck disability, pain, and BMI were associated with poorer functional and QoL outcomes. Functional ability occurred as a positive determinant of QoL. **Conclusions:** These results highlight the need for integrated management focusing on pain reduction, functional rehabilitation, and weight optimisation to improve quality of life in patients with cervical spondylosis.

## 1. Introduction

Cervical spondylosis is a highly ubiquitous degenerative condition characterised by intervertebral disc desiccation, facet arthropathy, and osteophyte formation, leading to neck pain, stiffness, and activity limitation across the adult lifespan [1,2]. In physically active populations and working-age adults, insistent neck symptoms are a major cause of health-related productivity loss and care seeking in sports and rehabilitation clinics [3]. Yet, routine clinical decision-making still depends on fragmented impressions of pain, impairment, and function rather than an integrated profile that links pain intensity, disability, performance, and QoL [4]. Although associations among pain, disability, function, and quality of life have been examined in general chronic neck pain populations, considerably fewer studies have evaluated these relationships specifically in clinically diagnosed cervical spondylosis. This condition involves degenerative changes that differ biomechanically and symptomatically from nonspecific neck pain, suggesting that determinants of functional performance and QoL may not follow identical patterns. Existing evidence rarely integrates pain, disability, functional capacity, body mass index (BMI), and cervical range-of-motion (ROM) within a single multivariable model. Therefore, identifying which routinely assessed clinical measures most strongly influence QoL in cervical spondylosis remains a relevant diagnostic and therapeutic gap.

Standardised patient-reported outcome measures (PROMs) allow that integration [5]. Pain intensity can be captured with the 0–10 Numeric Pain Rating Scale (NPRS), typically averaged across current, best, and worst pain over 24 h to reflect the patient’s typical level [6]. Activity limitation and work/participation restrictions are most assessed with the Neck Disability Index (NDI), an instrument with 10 items, scored 0–50, where higher scores indicate greater disability and categories map onto clinically meaningful severity bands [7,8]. Complementing condition-specific disability, the Patient-Specific Functional Scale (PSFS) provides an individualised index of performance by asking patients to rate up to three personally important activities from 0 (unable) to 10 (pre-injury level), with the mean score summarising functional capacity [9,10]. Because pain and disability reverberate beyond function to affect well-being, the McGill Quality of Life (MQOL) questionnaire captures global QoL alongside physical and existential/psychological facets, offering a broader perspective on patient-centred outcomes [11,12,13,14].

Even though each of these instruments is individually validated, fewer studies have examined their inter-relationships in cervical spondylosis specifically, where nociceptive drivers, kinematic constraints, and psychosocial responses may interact in unique ways. Understanding how pain intensity relates to disability, how disability relates to patient-chosen performance, and how all three relate to QoL can improve clinical reasoning, inform goal-setting, and identify targets for rehabilitation (e.g., whether reducing pain or improving specific activities yields larger QoL gains). Furthermore, basic impairment measures such as cervical ROM are routinely collected in musculoskeletal settings, but they are not consistently examined alongside PROMs to quantify their added explanatory value [15]. Although pain, disability, functional capacity, and quality of life have each been studied individually, there remains a lack of research integrating these dimensions into a unified analytical model for cervical spondylosis. This study addresses that gap by examining these interrelated constructs concurrently to understand their collective influence on quality of life. Cervical spondylosis affects pain, mobility, functional ability, and psychological well-being simultaneously, yet prior studies often evaluate these factors separately. Understanding their combined interaction is essential for improving patient-centred management.

Therefore, this cross-sectional study aims to (1) outline pain intensity, disability, functional performance, QoL, and key clinical measures in adults with cervical spondylosis; (2) quantify bivariate associations among NPRS, NDI, PSFS, MQOL, ROM, and BMI; and (3) build multivariable models to analyse independent associations of QoL, incorporating pain, disability, functional performance, body mass index, and adjusting for demographic factors (age, gender, profession). The objective of this study was to investigate how pain, disability, physical function, and BMI collectively relate to quality of life among adults with cervical spondylosis. It is hypothesised that higher pain intensity will be associated with higher disability, lower PSFS scores, and lower QoL; that NDI will show robust inverse correlations with PSFS and QoL than pain alone; and that ROM will explain additional variance in functional performance beyond pain and disability. By joining routinely collected clinic measures with validated PROMs, the findings aim to provide rational, evidence-informed guidance for prioritising assessments and interventions in sports and rehabilitation practice.

## 2. Materials and Methods

### 2.1. Study Design

This cross-sectional study was carried out amongst adult patients clinically diagnosed with cervical spondylosis and who attended private physiotherapy and rehabilitation clinics in Kosovo during 2023. As this sample was drawn from a single private physiotherapy centre, the demographic composition may not fully represent the broader cervical spondylosis population in Kosovo. The primary aim was to evaluate associations between pain intensity, disability, functional performance, BMI, and quality of life.

### 2.2. Participants

Participants were recruited using convenience sampling. The study included patients aged 18 years or more with a conventional diagnosis of cervical spondylosis who could understand and complete the study questionnaires.

### 2.3. Inclusion and Exclusion Criteria

Exclusion criteria comprised a history of cervical trauma, systemic inflammatory disorders, neurological deficits such as myelopathy or radiculopathy from other causes, recent cervical spine surgery, or any condition interfering with neck function or questionnaire comprehension.

### 2.4. Data Collection

Of the 150 screened patients, 111 participants aged from 18–70 years satisfied the inclusion eligibility criteria; data of 107 out of 111 participants were further analysed due to missing data. Ethical approval to conduct the research was obtained from the Kosovo Chamber of Physiotherapy (Prishtina, Kosovo) (Prot. No. 473), and written informed consent, along with a publication agreement, was obtained from all participants before the process of data collection. Each participant underwent standardised clinical and self-reported assessments during a single visit.

### 2.5. Demographic Variables

Demographic variables were collected to characterise the study population. These included age, gender, occupation, height (m), and weight (kg). Age was recorded in years and categorised into three groups (<40 years, 40–60 years, and >60 years) for descriptive and comparative analyses. Gender was recorded as male or female. BMI was calculated as weight in kilograms divided by height in metres squared. Occupation was recorded and subsequently classified according to physical work demands into physically active or sedentary occupation groups. These demographic variables were used to describe the sample characteristics and to support subgroup analyses.

### 2.6. Outcome Measures

Pain intensity was assessed using the 11-point NPR scale, where 0 represents no pain, and 10 indicates the worst pain imaginable. Participants reported their current as well as best and worst pain over the last 24 h, and the average of these three ratings was used as the overall pain intensity [13]. Functional disability related to neck pain was measured using the ten-item NDI [8]. With each item scored from 0–5, yielding a total score from 0 (no disability) to 50 (complete disability). For interpretation, total scores were categorised as: 0–4 (no disability), 5–14 (minor), 15–24 (moderate), 25–34 (severe), and ≥35 (complete disability). Functional performance was measured by asking each participant to list up to three daily activities affected by neck problems. Each activity was rated from 0 (unable to perform) to 10 (normal pre-injury performance). The mean of the three activity scores was used as the overall PSFS score [10]. The quality of life of patients was measured using the MQOL questionnaire, which evaluates physical symptoms (Part B), physical well-being (Part B-4), psychological and existential domains (Part C), and general perceived quality of life (Part A). Items are rated from 0 to 10, with higher scores representative of better well-being. The score from Part A was used as the global QoL index, while subscale means reflected domain-specific well-being. Objective cervical ROM was measured in degrees using a goniometer for flexion, extension, lateral flexion, and rotation.

### 2.7. Statistical Analysis

The data were processed using IBM SPSS Statistics version 24.0 (IBM Corp., Armonk, NY, USA) [16]. Computed variables in the analysis included the mean PSFS score, total and percentage NDI scores. Height and weight were recorded using standard stadiometer and digital scale measurements, and BMI was calculated as weight (kg) divided by height squared (m^2^). Age was categorised into three groups (<40, 40–60, >60 years), and professions were dichotomised into physically active versus sedentary based on work demands. Active professions included businesswoman, worker, teacher, information technology, policeman, whereas sedentary professions included retired, housewife, dentist, lawyer, economist, student, doctor. Data were further checked for completeness, and missing values were handled using listwise case deletion. Normality of continuous variables was calculated through descriptive analysis and the Shapiro–Wilk test. Descriptive statistics (mean ± SD, min-max) were reported for continuous data and frequencies (%) for categorical variables. Bivariate relationships among pain (NPRS), disability (NDI), function (PSFS), and quality of life (MQOL) were examined using Pearson’s correlation coefficients. Group differences were analysed using independent-samples *t*-tests (for gender and profession groups) and one-way ANOVA along with Tukey’s post hoc comparisons (for age groups). The regression analysis was not intended to develop an association model but to quantify the relative contribution of commonly assessed clinical variables in explaining quality-of-life variation. Multiple linear regression analyses were conducted to identify which variables independently correlated:

1. Model 1 (Quality of Life): Dependent = MQOL global score; explanatory variables = NDI, NPRS, PSFS, BMI, Age, Gender, Profession active, and ROM measures.
2. Model 2 (Functional Performance): Dependent = PSFS mean; explanatory variables = NDI, NPRS, Age, Gender, Profession active, BMI, and ROM measures.

Because this was a cross-sectional study, regression coefficients were interpreted as associations rather than causal effects. Multicollinearity was checked using the Variance Inflation Factor (VIF < 5 considered acceptable). The level of statistical significance was *p* < 0.05. SPSS 27.0.1.0 generated the plots that consisted of scatterplots with linear fits displaying the relationship of NDI vs. PSFS and NDI vs. QoL, in addition to having a boxplot displaying NDI and PSFS distribution by gender. Because objective cervical ROM measures were collected, an additional regression model was performed to examine whether ROM had independent associations with quality of life and functional performance.

## 3. Results

**Figure 1:**
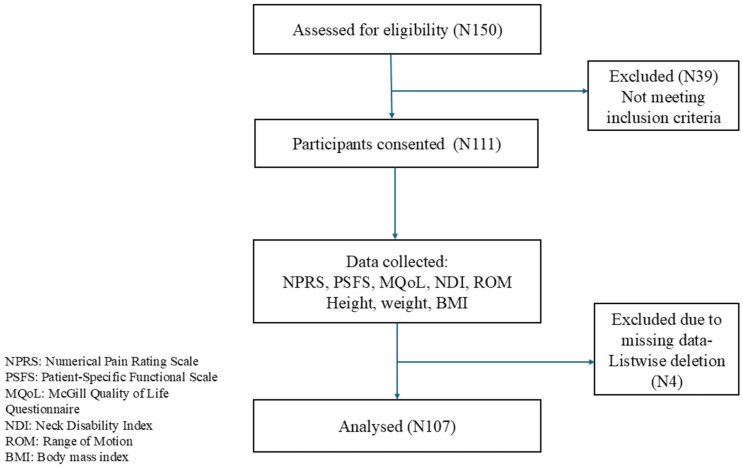
Flow Chart of the study.

This study included 150 patients with cervical spondylosis. The initial number of the sample has decreased from 150 to 111 as 39 patients were excluded; they did not meet the inclusion criteria for the study. 107 out of 111 participants were further analysed after listwise deletion due to missing data (Figure 1): 33 males (29.7%) and 78 females (70.3%). The average age of the participants was 51.51 ± 12.02 years with a range of 19–69 years (Table 1), 18 (16.8%) participants were younger than 40 years, 61 (57.0%) were aged between 40 and 60 years, and 28 (26.2%) were older than 60 years. Four participants (3.6%) had missing age data. Participants have a mean BMI of 27.97 ± 4.49 kg/m^2^.

The professional activity was distributed evenly, with 56 (50.5%) of the respondents having active jobs and 55 (49.5%) having sedentary jobs (Table 1). The sample on the NPRS showed a mean score of 7.05 ± 1.85, which shows predominantly moderate to severe levels of pain. The total score of the Neck Disability Index (NDI) was the average of 19.93 ± 7.78, equal to 39.86 ± 15.56 percentage, which indicates moderate levels of disability. Functional ability, measured through the PSFS mean score, was obtained as 4.01 ± 1.95, showing moderate activity limitation, whereas the MQOL (Part A) score averaged 5.47 ± 1.69, reflecting moderate perceived well-being.

Regarding physical measures, the mean cervical flexion was 47.48° ± 13.09°, extension 52.95° ± 13.30°, left side-bending 34.43° ± 7.85°, right side-bending 29.48° ± 8.01°, left rotation 51.88° ± 12.14°, and right rotation 52.99° ± 11.71°. The mean BMI of participants was 27.97 ± 4.49 kg/m^2^, suggesting that most participants were in the overweight category.

After descriptive results, the distribution of continuous variables was inspected using the Shapiro–Wilk test, as presented in Table 2. Among the primary outcome measures, NDI (*p* = 0.282) was the only variable found to be following a normal distribution. However, in contrast, pain intensity (NPRS), PSFS_mean, MQOL, and most ROM variables showed significant deviations from normality (*p* < 0.05).

The correlation analysis was then conducted, and results displayed several significant associations in the matrix between pain, disability, functional performance, and quality of life variables (Table 3). Pain intensity displayed a strong positive correlation with neck disability among participants (r = 0.532, *p* < 0.001). Pain was negatively correlated with quality of life (r = −0.462, *p* < 0.001), which signifies that increasing pain is associated with poorer well-being in this condition. Neck disability was found to be negatively correlated with both functional performance (r = −0.307, *p* = 0.001) and quality of life (r = −0.541, *p* < 0.001), showing that participants with higher disability scores had lesser functional ability and perceived quality of life. Functional performance demonstrated a strong positive relationship with QoL (r = 0.421, *p* < 0.001), indicating that the patients who functioned better in daily activities consistently reported higher levels of well-being.

Age, however, demonstrated a weak but significant correlation with neck disability (r = 0.245, *p* = 0.011), whereas a negative correlation with functional performance (r = −0.272, *p* = 0.005). Body mass index showed negative correlations with functional performance (r = −0.251, *p* = 0.009) and QoL (r = −0.240, *p* = 0.013). No significant correlations were found between range-of-motion parameters and pain or disability. However, intercorrelations among the ROM variables themselves were strong and positive (r = 0.3–0.6, *p* < 0.001), reflecting internal consistency within the mobility measures. These results showed that greater pain and disability are closely linked to poorer function and diminished quality of life, whereas better functional capacity corresponds to improved subjective well-being.

**Figure 2:**
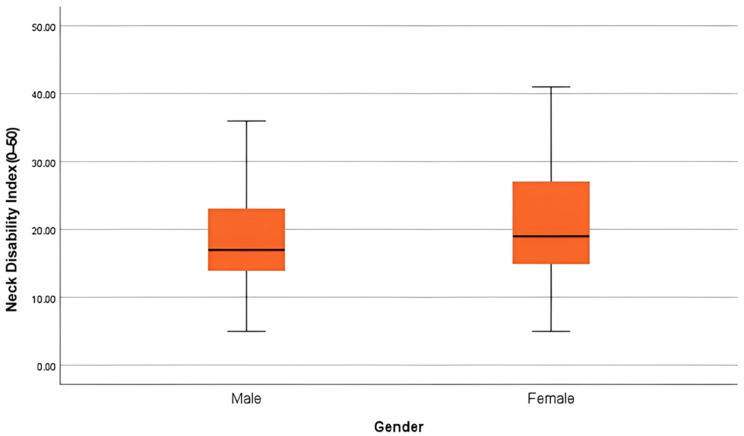
Boxplot of Neck Disability Index (0–50) by gender. Female participants showed a marginally higher median disability score, but variability and overlap suggest no significant gender effect.

Additionally, independent-samples *t*-tests were performed to study gender- and profession-based differences in pain intensity (NPRS), neck disability (NDI), functional ability (PSFS), and quality of life (MQOL). Female participants (M = 7.18, SD = 1.88) reported slightly higher pain scores than males (M = 6.73, SD = 1.76), but this difference was not observed to be statistically significant (t(109) = −1.18, *p* = 0.241). Similarly, no significant differences were observed between males and females in neck disability (t(109) = −1.38, *p* = 0.169), functional ability (t(109) = 0.54, *p* = 0.587), or quality of life (t(109) = 1.17, *p* = 0.244) (Table 4). The effect sizes (Cohen’s d = −0.24 to 0.24) for all gender comparisons were small, suggesting minimal clinical relevance of these differences. Figure 2 illustrates gender differences in Neck Disability Index (NDI) scores. The median disability level was slightly higher in females, but the distributions largely overlapped, confirming non-significant differences (*p* = 0.169).

**Figure 3:**
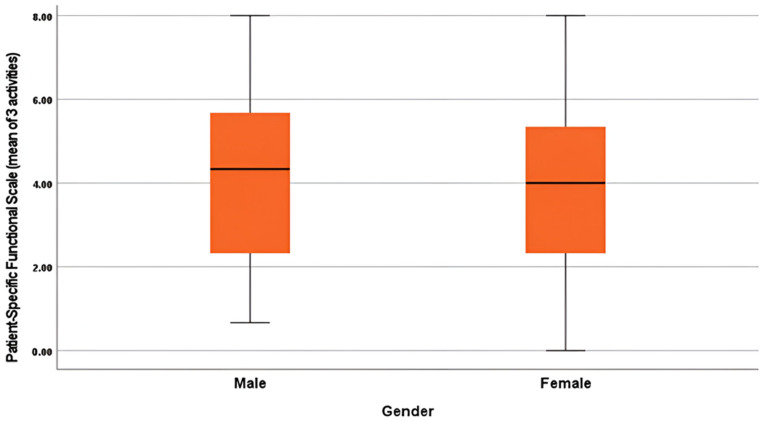
Boxplot of Patient-Specific Functional Scale (0–10) by gender. Males and females displayed similar functional ability levels, consistent with non-significant *t*-test results.

Regarding physical activity status, participants who were working in active professions (M = 7.05, SD = 1.76) and those with a sedentary form of profession (M = 7.04, SD = 1.95) verified nearly identical pain scores, with no significant difference (t(109) = 0.05, *p* = 0.961). Differences in neck disability (t(109) = 0.90, *p* = 0.368), functional ability (t(109) = −0.71, *p* = 0.478), and MQOL (t(109) = −0.92, *p* = 0.358) were also nonsignificant (Table 5). Effect size estimates for all comparisons were found to be negligible (Cohen’s d = −0.17 to 0.17), signifying that activity level based on the profession of an individual did not meaningfully influence pain, disability, or quality of life. Figure 3 displays the gender distribution of Patient-Specific Functional Scale (PSFS) scores. Both males and females demonstrated similar ranges and median values, supporting the statistical result of no significant difference (*p* = 0.587 *).

A one-way ANOVA was performed to examine whether outcomes differed across the three age categories (<40, 40–60, >60 years). The analysis indicated that none of the measured clinical variables, i.e., for neck disability (F(2, 104) = 1.69, *p* = 0.189), functional ability (F(2, 104) = 2.69, *p* = 0.072), or quality of life (F(2, 104) = 0.03, *p* = 0.974) varied significantly between age groups. Post hoc Tukey tests confirmed that none of the pairwise group differences were statistically significant (all *p* > 0.05). Levene’s test showed homogeneity of variances across all models, confirming that the assumptions for ANOVA were met (Table 6).

**Figure 4:**
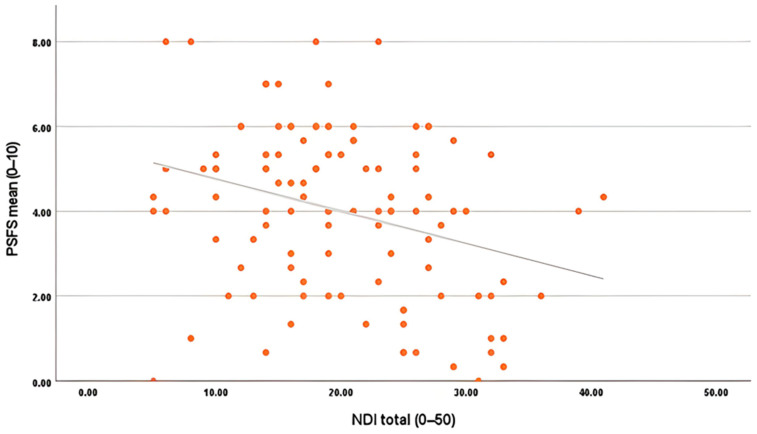
Scatter plot depicting the relationship between NDI (0–50) and PSFS (0–10). A negative correlation displayed that higher disability is linked with reduced functional ability.

After the multiple comparison using Tukey’s HSD test, two separate multiple linear regression models were constructed to identify explanatory variables of (a) overall quality of life (MQOL) and (b) functional ability (PSFS). Model 1, i.e., independent correlate of quality of Life (MQOL), showed that the regression model was found to be statistically significant (F(13, 93) = 7.04, *p* < 0.001), explaining 49.6% of the variance in MQOL scores (*R*^2^ = 0.496, Adjusted *R*^2^ = 0.426). An Adjusted *R*^2^ of 0.426 indicates a moderate level of explained variance, which is expected in multidimensional musculoskeletal conditions where psychological, social, and environmental determinants contribute to quality-of-life outcomes beyond clinical measures alone. Among the predictors, Neck Disability Index (NDI) (β = −0.366, *p* < 0.001), pain intensity (NPRS) (β = −0.252, *p* = 0.006), Patient-Specific Functional Scale (PSFS) (β = 0.238, *p* = 0.006), and Body Mass Index (BMI) (β = −0.298, *p* < 0.001) appeared as significant independent correlates that explained variance in quality of life (Table 7). Figure 4 shows the relationship between neck disability (NDI) and functional ability (PSFS). A clear negative linear trend is visible, indicating that patients with higher disability scores tend to report lower functional ability. The scatter is moderately dispersed, suggesting individual variation, yet the fitted line confirms a downward slope consistent with the regression output (β = −0.207).

Higher neck disability, pain, and BMI were associated with lower QoL, whereas higher functional ability (PSFS) was linked with better QoL. Age, gender, physical activity, and range of motion measures were not significant contributors. Collinearity diagnostics (condition index < 30) indicated no multicollinearity issues.

Model 2, i.e., Predictors of functional ability (PSFS), was also statistically significant (F(12, 94) = 2.45, *p* = 0.008), explaining 23.8% of the variance (*R*^2^ = 0.238, Adjusted *R*^2^ = 0.141).

**Figure 5:**
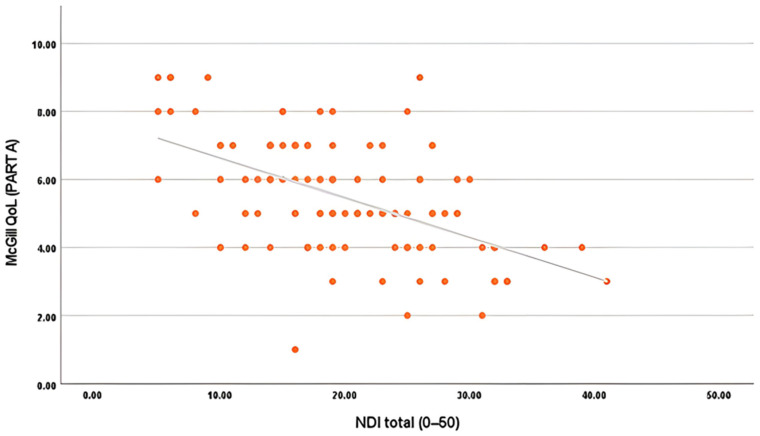
Scatter plot showing the association between NDI (0–50) and MQOL (Part A). A higher level of disability corresponds with a lower quality of life.

Among these, only Body Mass Index (BMI) (β = −0.230, *p* = 0.030) showed a significant negative association, indicating that higher BMI is associated with lower self-reported functional ability. Pain (NPRS), disability (NDI), and other demographic or biomechanical factors (age, gender, activity, or range of motion) were not significant in this model. Figure 5 depicts the relationship between NDI with quality of life (MQOL, Part A). The negative slope proves that increasing neck disability is strongly associated with lower QoL scores. This result aligns with the regression findings where NDI (β = −0.366, *p* < 0.001) was observed as one of the strongest independent correlates of QoL.

An additional regression model including cervical ROM variables (flexion, extension, side-bending, rotation) showed that none of the ROM parameters were statistically significant predictors of quality of life (all *p* > 0.28) (Table S1)

## 4. Discussion

This research was conducted to assess how pain, disability, functional performance, and body composition interact to influence the quality of life among patients with cervical spondylosis. Where previous research has observed isolated explanatory variables such as pain or range of motion, few studies have integrated both physical and psychosocial factors into a unified independent correlate model. This study, therefore, helps in filling that gap by analysing disability, function, and quality of life measures using validated tools.

The research states that neck disability and pain intensity show significant associations with quality of life, suggesting that physical discomfort extends its effects beyond mechanical limitations to influence the emotional and social well-being of the individual. The fact that the observed positive correlation between functional performance and QoL also suggests the significance of active involvement and personal independence in the process of remaining psychologically healthy as well. The effect of BMI added weight to the increasing awareness of the role of lifestyle, especially weight management, in musculoskeletal health. Also, there were no notable differences by gender or activity-based differences, and this showed that the burden of cervical disability can be widely consistent in different population subgroups, where a clinical emphasis should be placed on the severity of symptoms and rehabilitation that is unique to each patient. The descriptive results revealed that many of the representatives of this cohort are middle-aged women who moderate pain and disability. The average NDI score of about 20/50 was an indication of a moderate level of functional impairment that matches the populations of chronic neck pain. These findings are consistent with previous work showing that cervical pain conditions commonly present with overlapping pain, disability, and functional limitations [17,18]. Earlier studies have also emphasised that pain severity strongly influences disability and daily performance [19], which aligns with our results. However, unlike studies that included broader chronic neck pain populations, this analysis focuses specifically on cervical spondylosis, thereby clarifying how these routinely measured clinical variables behave within this diagnosis rather than in heterogeneous neck pain cohorts.

In a similar note, the mean result of the PSFS was 4, indicating that the participants encountered apparent but not serious difficulties on how to execute daily tasks, which is indicative of the functional demand in cervical spondylosis. This trend is seen in this research in congruence with other studies that have reported that NDI scores in patients with persistent neck pain are usually moderate (15–5/50), meaning that they have quantifiable impairments in daily functional tasks and not severe disability [17]. The average (approximately 5.5/10) score of the MQOL was also a confirmation of the multidimensional influence of cervical spondylosis, as both physical and psychosocial well-being are altered. Losses in cervical ROM, especially in flexion and lateral bending, were uniform with degenerative alterations in the cervix. The increased mean BMI (approximately 28 kg/m^2^) could also contribute to an increased mechanical load and postural stress on the cervical spine, which may aggravate the pain and disability. Similar results were noted in the previous studies that revealed that low QoL was observed when physical, emotional, and social domains were measured concurrently in cohorts of chronic neck pain [18]. Similarly, both limitations in flexion and lateral bending have been reported to be the features of degenerative changes in spondylosis, and higher BMI have been linked to increased spinal load and increased chances of neck pain in the affected patients [20,21], respectively. The pattern of moderate pain, reduced function, and diminished QoL is also comparable to previous studies on chronic neck pain and degenerative cervical changes [18,20]. However, the results from this research differ slightly from studies where cervical ROM showed stronger associations with disability [22]. In this study, ROM did not correlate meaningfully with either pain or disability. This suggests that, among individuals with cervical spondylosis, subjective disability and QoL may be more closely shaped by symptom severity and functional limitations than by isolated biomechanical restriction. The normal test showed that the NDI scores only exhibited a normal distribution pattern, whereas all the other continuous variables, such as pain intensity, physical functioning and quality of life, exhibited skewed patterns. This kind of deviation is largely anticipated in clinical cohorts, where patients are more likely to have a first-order deviation of around moderate-to-high severity of symptoms as opposed to being distributed perfectly symmetrically [23]. Likewise, the non-normality of the distributions of ROM and QoL variables is simply a byproduct of clinical heterogeneity of cervical spondylosis, wherein structural degeneration, variation in pain, and lifestyle differences among patients vary significantly.

Considering this heterogeneity, it is significant to use parametric and non-parametric statistical models to allow for the reliable interpretation and to reduce the bias generated by distributional assumptions. The moderate explanatory power of the regression model is consistent with the broader chronic pain literature, where QoL is influenced by diverse psychosocial and contextual factors outside biomechanical impairment [18,19]. Prior studies have highlighted that disability contributes more strongly to functional and psychosocial outcomes than pain intensity itself [24,25], which is reflected in our findings where NDI remained the strongest independent correlate. Therefore, although the constructs included in our model are interrelated, quantifying their relative contribution within a cervical spondylosis population adds clarity to how these routinely collected measures align with patient-reported well-being. The correlation analysis has indicated the strong interdependence between pain and disability, and functional performance and QoL among people affected by cervical spondylosis. The strong positive correlation between the level of pain and disability of the neck proved that the severity of pain was directly proportional to high functional disability, which is also congruent with earlier studies, which found pain severity to be a significant factor that contributes to functional impairment in acute neck pain cohorts [19]. Similarly, the fact that there is a strong negative relationship between pain and quality of life once again confirmed that patients who report higher amounts of severe pain are more likely to report poor psychological and physical well-being. The correlation between NDI scores and function and QoL of life was negative, and these results indicated that physical impairment due to cervical pathology is not only related to the ability to move but also to day-to-day functioning as well as subjective life satisfaction. Regression analysis further confirmed that cervical ROM did not independently contribute to quality of life or functional performance once pain, disability, BMI, and functional ability were entered into the model.

This agrees with the knowledge that cervical spondylosis has a multidimensional effect where the biomechanical dysfunction, perception of pain and psychosocial distress interplay to determine patient outcomes. These trends are consistent with the earlier data, which indicates that the level of pain has been one of the strongest associations of activity impairments and emotional suffering in patients with cervical spinal conditions [26]. Likewise, the observed negative relationship between NDI and both PSFS and QoL confirmed that greater disability coincides with poorer function and well-being, reinforcing earlier evidence that neck disability is associated with both physical and psychosocial outcomes [24].

The positive correlation between the functional scale and quality of life suggested that improved physical function contributes meaningfully to better subjective well-being, consistent with rehabilitation outcomes as suggested by literature emphasising functional recovery as a driver of life quality. The correlation results followed the trend reported in the rehabilitation study, showing that improvements in task-specific ability translate directly into enhanced perceived well-being [25]. This emphasises that functional recovery should be prioritised as a therapeutic goal.

Age and BMI showed weaker but noteworthy associations. The positive relationship between age and disability reflects the progressive degenerative changes and reduced musculoskeletal adaptability commonly observed in older adults. The negative correlations of BMI with function and quality of life suggest that higher body weight may exacerbate mechanical stress on the cervical spine and limit activity tolerance, indirectly affecting mental and social dimensions of health. The association between higher age and greater disability also reflects the age-related decline in musculoskeletal flexibility and adaptation capacity reported in previous cohort analyses [27]. The link between elevated BMI and reduced function or QoL validates earlier research showing that obesity contributes to chronic musculoskeletal strain and lower self-rated health in spinal conditions [28].

In contrast to this, the lack of significant relationships between range-of-motion parameters and pain or disability corroborates a previous report suggesting that cervical ROM does not necessarily correlate with perceived impairment because patients might compensate through modified movement strategies or pain avoidance behaviours [22].

Even though the parameters of the cervical ROM showed no significant correlations with pain or disability in the present study, the level of intercorrelations between them showed internal rationality across the physical assessment domains, which proved reliable measurement patterns. The lack of direct correlation with pain or disability might indicate compensatory movement practices, pain avoidance behaviour or chronicity and disease progression variations among the participants, none of which is linked to symptom severity. The findings point to the complex biopsychosocial character of cervical spondylosis, with mechanical restrictions not being considered sufficient to clarify the patient experience either functionally or psychologically. Together, the findings do not indicate that the reduction of pain in isolation is sufficient to significantly contribute to the well-being of patients. Rather, the management is to be carried out through the integrated approach that would care about functional rehabilitation, weight optimisation, and psychosocial assistance to achieve improved clinical results and the perceived quality of life. Subgroup analysis revealed no statistically significant gender, occupational, or age-associated differences. Although the pain and disability scores were a little higher among women, it was found that the differences between them are minimal and clinically unimportant. This finding is corroborated by past studies that, despite women recording higher levels of symptoms because of musculoskeletal disorders, this disparity may be explained by the fact that women are more sensitive to pain or rather by psychosocial factors and not functional impairment [29]. Similarly, occupational activity level did not influence outcomes, suggesting that sedentary work did not exacerbate symptoms as hypothesised, nor did active professions confer a protective advantage. This may indicate that ergonomic factors, posture habits, and individual pain coping mechanisms have a greater impact than the type of profession alone [30]. The absence of significant age-group differences hints that degenerative changes associated with ageing may not directly translate to worsened function or quality of life. It also supports the view that cervical spondylosis outcomes depend more on physical conditioning, pain management, and rehabilitation adherence of the individual than on chronological age. Together, these results suggest that the type of gender, profession, and age do not independently show association with symptom severity or functional limitation in cervical spondylosis. Instead, pain, disability, and psychosocial well-being appear to be interrelated and multifactorial, necessitating an all-inclusive treatment approach that focuses on individualised rehabilitation rather than only demographic characteristics.

This study has some strengths and limitations as well. First, the main strength of this study lies in its comprehensive inclusion of clinical, functional, and quality-of-life measures, which allowed a multidimensional understanding of cervical spondylosis outcomes. Second, the use of standardised and validated tools strengthens the reliability of the findings, and third, the regression model provides clear quantitative evidence of relationships. However, some limitations lie in the cross-sectional nature of this study, which prevents any inference of causality. Since all measures were self-reported, some degree of response bias cannot be excluded. Another limitation is that the single-centre sampling confines the generalizability of results across diverse populations.

This study should be extended to longitudinal design studies with repeated measures to have a better insight into the dynamics of pain, disability, and functional capacity over time in patients with cervical spondylosis/core condition. The inclusion of objective functional measures and post-rehabilitation follow-ups would be useful in clarifying the causal pathways and illuminating recovery pathways. Clinically, the current results suggest that functional capacity improvement that minimises the perceived disability has a stronger impact on the quality of life than demographic features. Rehabilitation programs must then emphasise functional objectives, specific pain management measures, as well as patient education directed at posture, pacing of activities and self-management in the long run. However, high BMI with a combination of lifestyle, exercise, and physiotherapy can be considered an added advantage that diminishes mechanical stress on the cervical spine and leads to better functional outcomes.

## 5. Conclusions

This research found disability, pain intensity, and BMI to be contributing to the lower quality of life of patients with cervical spondylosis, as compared to better functional ability, which emerged as protective. While the model explains a moderate proportion of QoL variance, this aligns with the complex, multidimensional nature of cervical spondylosis and reinforces the need to prioritise disability reduction and functional improvement in rehabilitation planning. It was also found that there are no gender or activity-based differences on which the rehabilitation plans were determined; hence, focus on functional enhancement and weight reduction as a personalised approach should be a priority instead of focusing on the difference in demographics. By integrating multiple clinical and patient-reported measures within a single model, this study provides a consolidated view of how disability, pain, function, and BMI collectively shape quality of life—a perspective that expands upon existing single-dimension studies. These findings are in favour of a holistic, functional-based rehabilitative strategy that goes beyond symptom management to encompass lifestyle change and psychosocial well-being, hence giving a more holistic channel of enhancing clinical outcomes in cervical spondylosis.

## Figures and Tables

**Table 1:** Descriptive characteristics of participants with cervical spondylosis.

	N	Minimum	Maximum	Mean	SD
Age	107	19	69	51.51	12.016
NPRS	111	3	10	7.05	1.851
NDI (0–50)	111	5.00	41.00	19.9279	7.77842
NDI (%)	111	10.00	82.00	39.8559	15.55684
PSFS (mean of 3 activities)	111	0.00	8.00	4.0060	1.95297
MQOL (PART A overall)	111	1.00	9.00	5.4685	1.69395
Flexion	111	20	80	47.48	13.086
Extension	111	18	70	52.95	13.304
SideB_left	111	18	52	34.43	7.846
SideB_right	111	12	58	29.48	8.010
Rotation_left	111	10	80	51.88	12.135
Rotation_right	111	26	87	52.99	11.714
Body Mass Index (kg/m^2^)	111	0.00	39.06	27.9670	4.48897
Valid N (listwise)	107				

SD = Standard deviation; NDI = Neck Disability Index (score range 0–50); PSFS = Patient-Specific Functional Scale; MQOL = McGill Quality of Life Questionnaire; SideB = Side Bending.

**Table 2:** Kolmogorov–Smirnov and Shapiro–Wilk test results for assessing the normality of variables.

	Kolmogorov–Smirnov ^a^			Shapiro–Wilk		
	Statistic	df	Sig.	Statistic	df	Sig.
Age	0.120	107	0.001	0.930	107	0.000
NPRS	0.152	107	0.000	0.946	107	0.000
NDI (0–50)	0.096	107	0.016	0.985	107	0.282
PSFS (mean of 3 activities)	0.112	107	0.002	0.969	107	0.013
MQOL (PART A overall)	0.143	107	0.000	0.964	107	0.005
Body Mass Index (kg/m^2^)	0.073	107	0.200 *	0.858	107	0.000
Flexion	0.147	107	0.000	0.962	107	0.004
Extension	0.179	107	0.000	0.906	107	0.000
SideB_left	0.148	107	0.000	0.952	107	0.001
SideB_right	0.172	107	0.000	0.954	107	0.001
Rotation_left	0.134	107	0.000	0.957	107	0.002
Rotation_right	0.122	107	0.001	0.966	107	0.008

Sig. = Significance; *p* < 0.05 considered statistically significant; NPRS = Numeric Pain Rating Scale; NDI = Neck Disability Index; PSFS = Patient-Specific Functional Scale; MQOL = McGill Quality of Life Questionnaire; SideB = Side Bending; * = This is a lower bound of the true significance; a = Lilliefors Significance Correction.

**Table 3:** Correlation Coefficients among variables.

		NPRS	Neck Disability Index (0–50)	Patient-Specific Functional Scale (Mean of 3 Activities)	MQOL (PART A Overall)	Body Mass Index (kg/m^2^)	Age	Flexion	Extension	SideB_Left	SideB_Right	Rotation_Left	Rotation_Right
NPRS	Pearson Correlation	1	0.532	−0.177	−0.462	−0.105	0.020	−0.047	−0.073	−0.109	−0.017	−0.109	−0.062
	Sig. (2-tailed)		0.000	0.069	0.000	0.283	0.840	0.634	0.452	0.263	0.866	0.264	0.525
NDI (0–50)	Pearson Correlation	0.532	1	−0.307	−0.541	−0.001	0.245	−0.032	−0.044	−0.113	−0.137	−0.165	−0.135
	Sig. (2-tailed)	0.000		0.001	0.000	0.988	0.011	0.745	0.654	0.247	0.161	0.089	0.164
PSFS (mean of 3 activities)	Pearson Correlation	−0.177	−0.307	1	0.421	−0.251	−0.272	−0.046	−0.042	−0.104	0.063	−0.036	−0.018
	Sig. (2-tailed)	0.069	0.001		0.000	0.009	0.005	0.636	0.664	0.285	0.517	0.710	0.853
MQOL (PART A overall)	Pearson Correlation	−0.462	−0.541	0.421	1	−0.240	−0.086	−0.089	0.056	0.102	0.045	0.055	0.039
	Sig. (2-tailed)	0.000	0.000	0.000		0.013	0.381	0.362	0.567	0.294	0.643	0.570	0.691
Body Mass Index (kg/m^2^)	Pearson Correlation	−0.105	−0.001	−0.251	−0.240	1	0.408	−0.041	−0.093	−0.056	−0.058	0.000	−0.191
	Sig. (2-tailed)	0.283	0.988	0.009	0.013		0.000	0.672	0.340	0.568	0.550	0.999	0.049
Age	Pearson Correlation	0.020	0.245	−0.272	-0.086	0.408	1	−0.273	−0.040	−0.159	−0.292	−0.025	−0.175
	Sig. (2-tailed)	0.840	0.011	0.005	0.381	0.000		0.005	0.681	0.102	0.002	0.798	0.072
Flexion	Pearson Correlation	−0.047	−0.032	−0.046	-0.089	−0.041	−0.273	1	0.415	0.163	0.460	0.379	0.341
	Sig. (2-tailed)	0.634	0.745	0.636	0.362	0.672	0.005		0.000	0.093	0.000	0.000	0.000
Extension	Pearson Correlation	−0.073	−0.044	−0.042	0.056	−0.093	−0.040	0.415	1	0.305	0.365	0.423	0.262
	Sig. (2-tailed)	0.452	0.654	0.664	0.567	0.340	0.681	0.000		0.001	0.000	0.000	0.006
SideB_left	Pearson Correlation	−0.109	−0.113	−0.104	0.102	−0.056	−0.159	0.163	0.305	1	0.538	0.332	0.439
	Sig. (2-tailed)	0.263	0.247	0.285	0.294	0.568	0.102	0.093	0.001		0.000	0.000	0.000
SideB_right	Pearson Correlation	−0.017	−0.137	0.063	0.045	−0.058	−0.292	0.460	0.365	0.538	1	0.345	0.415
	Sig. (2-tailed)	0.866	0.161	0.517	0.643	0.550	0.002	0.000	0.000	0.000		0.000	0.000
Rotation_left	Pearson Correlation	−0.109	−0.165	−0.036	0.055	0.000	−0.025	0.379	0.423	0.332	0.345	1	0.686
	Sig. (2-tailed)	0.264	0.089	0.710	0.570	0.999	0.798	0.000	0.000	0.000	0.000		0.000
Rotation_right	Pearson Correlation	−0.062	−0.135	−0.018	0.039	−0.191	−0.175	0.341	0.262	0.439	0.415	0.686	1
	Sig. (2-tailed)	0.525	0.164	0.853	0.691	0.049	0.072	0.000	0.006	0.000	0.000	0.000	

Listwise N = 107. NPRS = Numeric Pain Rating Scale; NDI = Neck Disability Index; PSFS = Patient-Specific Functional Scale; MQOL = McGill Quality of Life Questionnaire; SideB = Side Bending

**Table 4:** Independent samples *t*-test comparing pain, disability, function, and QoL between male and female participants.

		Levene’s Test for Equality of Variances		*t*-Test for Equality of Means						
		F	Sig.	T	df	Sig. (2-Tailed)	Mean Difference	SE Difference	95% Confidence Interval of the Difference	
									Lower	Upper
NPRS	Equal variances assumed	0.080	0.778	−1.179	109	0.241	−0.452	0.384	−1.213	0.308
	Equal variances not assumed.			−1.214	64.469	0.229	−0.452	0.373	−1.197	0.292
NDI (0–50)	Equal variances assumed	0.427	0.515	−1.384	109	0.169	−2.22611	1.60861	−5.41431	0.96210
	Equal variances not assumed.			−1.411	63.082	0.163	−2.22611	1.57712	−5.37765	0.92544
PSFS (mean of 3 activities)	Equal variances assumed.	0.010	0.920	0.544	109	0.587	0.22145	0.40686	−0.58494	1.02783
	Equal variances not assumed.			0.547	60.935	0.587	0.22145	0.40502	−0.58845	1.03134
MQOL (PART A overall)	Equal variances assumed	0.334	0.564	1.172	109	0.244	0.41142	0.35117	−0.28459	1.10744
	Equal variances not assumed.			1.170	60.086	0.247	0.41142	0.35178	−0.29223	1.11507

SE = Standard error; Sig. = Significance; *p* < 0.05 considered statistically significant; NPRS = Numeric Pain Rating Scale; NDI = Neck Disability Index; PSFS = Patient-Specific Functional Scale; MQOL = McGill Quality of Life Questionnaire.

**Table 5:** Independent samples *t*-test comparing pain, disability, function, and QoL between active and sedentary occupations.

		Levene’s Test for Equality of Variances		*t*-Test for Equality of Means						
		F	Sig.	T	df	Sig. (2-Tailed)	Mean Difference	SD Difference	95% Confidence Interval of the Difference	
									Lower	Upper
NPRS	Equal variances assumed	0.551	0.459	0.049	109	0.961	0.017	0.353	−0.682	0.717
	Equal variances not assumed			0.049	107.445	0.961	0.017	0.353	−0.683	0.718
NDI (0–50)	Equal variances assumed	0.259	0.612	0.903	109	0.368	1.33474	1.47789	−1.59439	4.26387
	Equal variances not assumed.			0.903	108.837	0.369	1.33474	1.47816	−1.59498	4.26446
PSFS (mean of 3 activities)	Equal variances assumed	5.156	0.025	−0.712	109	0.478	−0.26439	0.37159	−1.00087	0.47208
	Equal variances not assumed.			−0.713	106.370	0.478	−0.26439	0.37099	−0.99990	0.47111
MQOL (PART A overall)	Equal variances assumed	2.201	0.141	−0.922	109	0.358	−0.29675	0.32180	−0.93454	0.34104
	Equal variances not assumed.			−0.923	108.613	0.358	−0.29675	0.32157	−0.93412	0.34061

SD = Standard deviation; SE = Standard error; Sig. = Significance; *p* < 0.05 considered statistically significant; NPRS = Numeric Pain Rating Scale; NDI = Neck Disability Index; PSFS = Patient-Specific Functional Scale; MQOL = McGill Quality of Life Questionnaire.

**Table 6:** One-Way ANOVA comparing pain, disability, function, and QoL across.

		Sum of Squares	df	Mean Square	F	Sig.
NDI (0–50)	Between Groups	206.302	2	103.151	1.693	0.189
	Within Groups	6336.352	104	60.926		
	Total	6542.654	106			
PSFS (mean of 3 activities)	Between Groups	20.476	2	10.238	2.694	0.072
	Within Groups	395.235	104	3.800		
	Total	415.711	106			
MQOL (PART A overall)	Between Groups	0.157	2	0.078	0.026	0.974
	Within Groups	312.199	104	3.002		
	Total	312.355	106			

Age Groups (<40, 40–60, >60 Years); NDI = Neck Disability Index; PSFS = Patient-Specific Functional Scale; MQOL = McGill Quality of Life Questionnaire.

**Table 7:** Post Hoc pairwise comparisons using Tukey’s HSD test to examine differences between the three age groups.

Dependent Variable	Age Group (I)	Age Group (J)	Mean Difference (I–J)	SE	Sig.	95% Confidence Interval	
						Lower Bound	Upper Bound
NDI (0–50)	<40	40–60	−2.78415	2.09370	0.382	−7.7624	2.1941
		>60	−4.33333	2.35812	0.162	−9.9403	1.2737
	40–60	<40	2.78415	2.09370	0.382	−2.1941	7.7624
		>60	−1.54918	1.78178	0.661	−5.7858	2.6874
	>60	<40	4.33333	2.35812	0.162	−1.2737	9.9403
		40–60	1.54918	1.78178	0.661	−2.6874	5.7858
PSFS (mean of 3 activities)	<40	40–60	0.98725	0.52291	0.147	−0.2561	2.2306
		>60	1.34127	0.58895	0.063	−0.0591	2.7416
	40–60	<40	−0.98725	0.52291	0.147	−2.2306	0.2561
		>60	0.35402	0.44500	0.707	−0.7041	1.4121
	>60	<40	−1.34127	0.58895	0.063	−2.7416	0.0591
		40–60	−0.35402	0.44500	0.707	−1.4121	0.7041
MQOL (PART A overall)	<40	40–60	0.03461	0.46474	0.997	−1.0704	1.1396
		>60	−0.05556	0.52343	0.994	−1.3001	1.1890
	40–60	<40	−0.03461	0.46474	0.997	−1.1396	1.0704
		>60	−0.09016	0.39550	0.972	−1.0306	0.8502
	>60	<40	0.05556	0.52343	0.994	−1.1890	1.3001
		40–60	0.09016	0.39550	0.972	−0.8502	1.0306

SE = Standard error; Sig. = Significance; *p* < 0.05 considered statistically significant; NDI = Neck Disability Index; PSFS = Patient-Specific Functional Scale; MQOL = McGill Quality of Life Questionnaire.

## Data Availability

Data are available from the main author upon reasonable request.

## Supplementary Materials

The following supporting information can be downloaded at: https://www.mdpi.com/article/10.3390/jcm15010094/s1, Table S1: Multiple regression models including cervical ROM variables as predictors of (a) Quality of Life (PART A) and (b) Functional Performance (PSFS).

## Abbreviations

The following abbreviations are used in this manuscript:

QoL	Quality of Life
NPRS	Numerical Pain Rating Scale
NDI	Neck Disability Index
PSFS	Patient-Specific Functional Scale
BMI	Body mass index
MQOL	McGill Quality of Life Questionnaire
ROM	Range of Motion
PROMs	Standardised patient-reported outcome measures

## References

[1] Margetis K., Tadi P. (2025). Cervical Spondylosis. StatPearls.

[2] Kelly J.C., Groarke P.J., Butler J.S., Poynton A.R., O’Byrne J.M. (2012). The Natural History and Clinical Syndromes of Degenerative Cervical Spondylosis. Adv. Orthop..

[3] Cheung J., Kajaks T., MacDermid J.C. (2013). The Relationship Between Neck Pain and Physical Activity. Open Orthop. J..

[4] Hadi M.A., McHugh G.A., Closs S.J. (2019). Impact of Chronic Pain on Patients’ Quality of Life: A Comparative Mixed-Methods Study. J. Patient Exp..

[5] Kasturi S., Price L.L., LeClair A., Patel N., Shetty S., Sheira D., Weber S., Curtis D., Nowell W.B., Salmon J. (2022). Clinical Integration of Patient-Reported Outcome Measures to Enhance the Care of Patients with SLE: A Multi-Centre Prospective Cohort Study. Rheumatology.

[6] Nugent S.M., Lovejoy T.I., Shull S., Dobscha S.K., Morasco B.J. (2021). Associations of Pain Numeric Rating Scale Scores Collected during Usual Care with Research Administered Patient Reported Pain Outcomes. Pain Med..

[7] Jorritsma W., de Vries G.E., Dijkstra P.U., Geertzen J.H.B., Reneman M.F. (2012). Neck Pain and Disability Scale and Neck Disability Index: Validity of Dutch Language Versions. Eur. Spine J..

[8] Vernon H., Mior S. (1991). The Neck Disability Index: A Study of Reliability and Validity. J. Manip. Physiol. Ther..

[9] Evensen J., Soberg H.L., Sveen U., Hestad K.A., Bronken B.A. (2020). The Applicability of the Patient-Specific Functional Scale (PSFS) in Rehabilitation for Patients with Acquired Brain Injury (ABI)—A Cohort Study. J. Multidiscip. Healthc..

[10] Westaway M.D., Stratford P.W., Binkley J.M. (1998). The Patient-Specific Functional Scale: Validation of Its Use in Patients with Neck Dysfunction. J. Orthop. Sports Phys. Ther..

[11] Serrano P.V., Serrano G.B., Torres I.L.S., Graudner R.R., Caumo W. (2020). The McGill Quality of Life Questionnaire-Revised (MQOL-R). Psychometric Properties and Validation of a Brazilian Version on Palliative Care Patients: A Cross-Sectional Study. Health Qual. Life Outcomes.

[12] Cohen S.R., Russell L.B., Leis A., Shahidi J., Porterfield P., Kuhl D.R., Gadermann A.M., Sawatzky R. (2019). More Comprehensively Measuring Quality of Life in Life-Threatening Illness: The McGill Quality of Life Questionnaire—Expanded. BMC Palliat. Care.

[13] McCaffery M., Beebe A. (1989). The Numeric Pain Rating Scale Instructions. Pain Clinic Manual for Nursing Practice.

[14] Cohen S.R., Mount B.M., Strobel M.G., Bui F. (1995). The McGill Quality of Life Questionnaire: A Measure of Quality of Life Appropriate for People with Advanced Disease. A Preliminary Study of Validity and Acceptability. Palliat. Med..

[15] Parazza S., Vanti C., O’Reilly C., Villafañe J.H., Tricás Moreno J.M., Estébanez De Miguel E. (2014). The Relationship between Cervical Flexor Endurance, Cervical Extensor Endurance, VAS, and Disability in Subjects with Neck Pain. Chiropr. Man. Ther..

[16] IBM Corp (2016). IBM SPSS Statistics for Windows.

[17] Torad A.A., Ahmed M.M., Elabd O.M., El-Shamy F.F., Alajam R.A., Amin W.M., Alfaifi B.H., Elabd A.M. (2024). Identifying Predictors of Neck Disability in Patients with Cervical Pain Using Machine Learning Algorithms: A Cross-Sectional Correlational Study. J. Clin. Med..

[18] Bagwan H.A., Varadharajulu D.G. (2024). Analysis Of Quality of Life in Chronic Neck Pain Patients. Afr. J. Biomed. Res..

[19] MacDermid J.C., Walton D.M., Bobos P., Lomotan M., Carlesso L. (2016). A Qualitative Description of Chronic Neck Pain Has Implications for Outcome Assessment and Classification. Open Orthop. J..

[20] Lindenmann S., Tsagkaris C., Farshad M., Widmer J. (2022). Kinematics of the Cervical Spine Under Healthy and Degenerative Conditions: A Systematic Review. Ann. Biomed. Eng..

[21] Lucha-López M.O., Hidalgo-García C., Monti-Ballano S., Márquez-Gonzalvo S., Ferrández-Laliena L., Müller-Thyssen-Uriarte J., Lucha-López A.C. (2023). Body Mass Index and Its Influence on Chronic Low Back Pain in the Spanish Population: A Secondary Analysis from the European Health Survey (2020). Biomedicines.

[22] Vishal K., Walkay A., Huixin T., Bhat V.S., Neelapala Y.V.R. (2023). The Relationship between Cervical Spine Range of Motion and Postural Sway in Mechanical Neck Pain: A Cross-Sectional Study. Hong Kong Physiother. J..

[23] Kamath A., Poojari S., Varsha K. (2025). Assessing the Robustness of Normality Tests under Varying Skewness and Kurtosis: A Practical Checklist for Public Health Researchers. BMC Med. Res. Methodol..

[24] Wibault J., Öberg B., Dedering Å., Löfgren H., Zsigmond P., Persson L., Peolsson A. (2014). Individual Factors Associated with Neck Disability in Patients with Cervical Radiculopathy Scheduled for Surgery: A Study on Physical Impairments, Psychosocial Factors, and Life Style Habits. Eur. Spine J..

[25] Prasad L., Fredrick J., Aruna R. (2021). The Relationship between Physical Performance and Quality of Life and the Level of Physical Activity among the Elderly. J. Educ. Health Promot..

[26] Lam K., Peolsson A., Soldini E., Löfgren H., Wibault J., Dedering Å., Öberg B., Zsigmond P., Barbero M., Falla D. (2021). Larger Pain Extent Is Associated with Greater Pain Intensity and Disability but Not with General Health Status or Psychosocial Features in Patients with Cervical Radiculopathy. Medicine.

[27] Azzolino D., Spolidoro G.C.I., Saporiti E., Luchetti C., Agostoni C., Cesari M. (2021). Musculoskeletal Changes Across the Lifespan: Nutrition and the Life-Course Approach to Prevention. Front. Med..

[28] Menoth Mohan D., Al Anouti F., Kohli N., Khalaf K. (2025). Association of Obesity with Musculoskeletal Health and Functional Mobility in Females—A Systematic Review. Int. J. Obes..

[29] Stieger A., Asadauskas A., Luedi M.M., Andereggen L. (2025). Women’s Pain Management Across the Lifespan—A Narrative Review of Hormonal, Physiological, and Psychosocial Perspectives. J. Clin. Med..

[30] Nygaard N.-P.B., Thomsen G.F., Rasmussen J., Skadhauge L.R., Gram B. (2022). Ergonomic and Individual Risk Factors for Musculoskeletal Pain in the Ageing Workforce. BMC Public Health.

