# Supplementary material for: A Cross-Sectional Analysis of Pain, Neck Disability, Functional Performance, and Quality of Life in Patients with Cervical Spondylosis
Source: J Clin Med. 2025 Dec 23;15(1):94. doi: 10.3390/jcm15010094 (PMC12786512; doi:10.3390/jcm15010094)
Supplement: Supplementary file 1 [file jcm-15-00094-s001.zip › jcm-4023770-supplementary.pdf]

Supplementary Tables:

Table S1 Multiple regression models including cervical ROM variables as predictors of (a) Quality of Life (PART A) and (b) Functional Performance (PSFS).

a)

Model		Unstandardized Coefficients		Standardized Coefficients	t	Sig.
		B	Std. Error	Beta		
1	(Constant)	9.455	1.67		5.662	0
	NRS_ pain	-0.232	0.083	-0.252	-2.795	0.006
	Flexion	-0.01	0.013	-0.075	-0.765	0.446
	Extention	0.002	0.012	0.016	0.178	0.859
	SideB_left	0.023	0.021	0.104	1.07	0.287
	SideB_right	0.003	0.022	0.016	0.151	0.88
	Rotation_left	0.002	0.016	0.017	0.153	0.878
	Rotation_right	-0.013	0.016	-0.09	-0.791	0.431
	Patient-Specific Functional Scale (mean of 3 activities)	0.206	0.073	0.238	2.822	0.006
	Neck Disability Index (0–50)	-0.08	0.021	-0.366	-3.802	0
	Body Mass Index (kg/m^2)	-0.112	0.033	-0.298	-3.401	0.001
	Age	0.025	0.013	0.174	1.877	0.064
	Gender	-0.04	0.324	-0.011	-0.123	0.903
	Physical activity type (1=Active, 2=Sedentary)	0.234	0.286	0.069	0.819	0.415

a Dependent Variable: PART A

b)

Model		Unstandardized Coefficients		Standardized Coefficients	t	Sig.
		B	Std. Error	Beta		
1	(Constant)	11.737	2.021		5.807	0
	NRS_ pain	-0.13	0.116	-0.122	-1.115	0.268
	Flexion	-0.024	0.018	-0.155	-1.3	0.197
	Extention	-0.003	0.016	-0.023	-0.208	0.836

SideB_left	-0.059	0.029	-0.232	-1.995	0.049
SideB_right	0.049	0.031	0.198	1.582	0.117
Rotation_left	0.003	0.022	0.019	0.139	0.89
Rotation_right	-0.011	0.023	-0.065	-0.47	0.639
Neck Disability Index (0–50)	-0.052	0.029	-0.207	-1.787	0.077
Body Mass Index (kg/m^2)	-0.1	0.045	-0.23	-2.198	0.03
Age	-0.028	0.018	-0.17	-1.511	0.134
Gender	0.112	0.457	0.026	0.245	0.807
Physical activity type (1=Active, 2=Sedentary)	0.352	0.402	0.089	0.875	0.384

a Dependent Variable: Patient-Specific Functional Scale (mean of 3 activities)
